# Paeonol Protects Memory after Ischemic Stroke via Inhibiting ***β***-Secretase and Apoptosis

**DOI:** 10.1155/2012/932823

**Published:** 2012-03-13

**Authors:** Shan-Yu Su, Chin-Yi Cheng, Tung-Hu Tsai, Ching-Liang Hsieh

**Affiliations:** ^1^Department of Chinese Medicine, China Medical University Hospital, Taichung 40447, Taiwan; ^2^School of Post-Baccalaureate Chinese Medicine, College of Chinese Medicine, China Medical University, Taichung 40402, Taiwan; ^3^Graduate Institute of Acupuncture Science, College of Chinese Medicine, China Medical University, 91 Hsueh-Shih Road, Taichung 40402, Taiwan; ^4^Institute of Traditional Medicine, National Yang-Ming University, Taipei 11221, Taiwan

## Abstract

Poststroke dementia commonly occurs following stroke, with its pathogenesis related to *β*-amyloid production and apoptosis. The present study evaluate the effects of paeonol, one of the phenolic phytochemicals isolated from the Chinese herb *Paeonia suffruticosa* Andrews (MC), on protection from memory loss after ischemic stroke in the subacute stage. Rats were subjected to transient middle cerebral artery occlusion (tMCAo) with 10 min of ischemia. The data revealed that paeonol recovered the step-through latency in the retrieval test seven days after tMCAo, but did not improve the neurological deficit induced by tMCAo. Levels of Amyloid precursor protein (APP)- and beta-site APP cleaving enzyme (BACE; *β*-secretase)-immunoreactive cells, and terminal deoxynucleotidyl transferase-mediated dUTP-biotin nick end labeling (TUNEL)-positive cells decreased in the paeonol-administered group. Western blotting revealed decreased levels of Bax protein in mitochondria and apoptosis-inducing factor (AIF) in cytosol following paeonol treatment. In conclusion, we speculate that paeonol protected memory after ischemic stroke via reducing APP, BACE, and apoptosis. Supression the level of Bax and blocking the release of AIF into cytosol might participate in the anti-apoptosis provided by paeonol.

## 1. Introduction

Cognitive impairment is a commonly occurring sequela following stroke and is the second leading cause of dementia in the elderly, after Alzheimer's disease [1, 2]. The majority of stroke survivors suffer from various cognitive dysfunctions, including dementia (which exists in 25% of stroke patients), linked with disability, poor functional outcome, and life dissatisfaction [3]. With recent improvements in the medical treatment of stroke, survival rates have increased, and the prevention and treatment of vascular cognitive impairment and vascular dementia after stroke have become increasingly important [3].

Although there are differences in the causes of the diseases, poststroke dementia shares common mechanisms with Alzheimer's disease, including the augmentation of *β*-amyloid production and tau protein phosphorylation [4, 5]. Following ischemic episode, the expression of amyloid precursor protein (APP) upregulates *β*-amyloid oligomers in the brain's extracellular spaces [6–8], and amyloid precursor protein production increases in astrocytes [9]. Then, the interactions between *β*-amyloid and several factors, including apolipoproteins, presenilins, tau protein, *α*-synuclein, inflammation factors, and neuronal survival/death decisions in the brain, contribute to ischemic brain degeneration, leading to white matter damage and neuronal cell death [5, 7]. Excepting necrotic cell death which happens within minutes, neuronal death (including apoptosis) commences several hours after ischemic stroke and lasts several days [10]. Therapies which can salvage neuronal cells from apoptosis in ischemic penumbra might improve patient outcomes after ischemic stroke [11].

Paeonol (2′-hydroxy-4′-methoxyacetophenone) is one of the phenolic phytochemicals isolated from the Chinese herb *Paeonia suffruticosa* Andrews (MC), and widely consumed as a nutrient supplement in a Chinese medical formula which had the third highest sales volumes in Beijing during 2007 and 2009 [12]. In an animal study, paeonol attenuated neurotoxicity and ameliorated cognitive impairment induced by d-galactose [13]. The present group has previously demonstrated that paeonol reduced the infarct area in a transient middle cerebral artery occlusion (tMCAo) rat model [14]. It also attenuated oxidative stress-induced APP expression in a cell model [15].

To investigate the validity of paeonol in protection of memory after stroke, rat model of tMCAo was used. Neurological status and memory was tested seven days after tMCAo. Levels of APP, beta-site APP cleaving enzyme (BACE), and apoptosis in brain tissue were evaluated. Then, the levels of proteins that participate in apoptotic intrinsic and extrinsic pathways were also measured. The data revealed that paeonol protected memory after ischemic stroke via reducing APP, BACE, and apoptosis. Suppression of level of mitochondrial Bax and cytosolic AIF might also participate in the protective effect.

## 2. Materials and Methods

### 2.1. Animals and Chemicals

All experimental procedures were performed on adult male Sprague-Dawley rats, weighing 300 to 350 g, according to the guidelines approved by the Care and Use of Laboratory Animals Committee of China Medical University. Adequate measures were taken to minimize animals' pain or discomfort. Rats were housed under light-dark—(12 h/12 h) and room temperature—controlled conditions.

Paeonol was isolated and purified from the root bark of *Paeonia suffruticosa* as described previously [14]. Freshly prepared paeonol was first dissolved in tetraglycol and then diluted in phosphate-buffered saline (PBS; 137 mM NaCl, 1.4 mM KH_2_PO_4_, 4.3 mM Na_2_HPO_4_, 2.7 mM KCl, pH 7.2) to reach a final concentration of 2 mg/mL in a 5% solution of  tetraglycol in PBS. Chloral hydrate (Merck, Darmstadt, Germany) was dissolved in water to a stock concentration of 400 mg/mL.

### 2.2. Occlusion Model

Ischemia was induced via intraluminal suture occlusion of the middle cerebral artery (MCA) as described previously [16]. Briefly, rats were anesthetized with chloral hydrate (400 mg/kg, i.p.). The right common carotid artery (CCA) and internal carotid artery (ICA) were exposed via a midline incision in the neck. The pterygopalatine artery was ligated close to its origin. A 3/0 nylon filament suture, blunted at the tip by a flame and coated with poly-L-lysine (Sigma, USA), was advanced from the right external carotid artery through the CCA and up to the ICA for a distance of 20 to 25 mm to block the origin of the MCA. After 10 min of ischemia, the nylon suture was removed to allow reperfusion.

### 2.3. Grouping and Experiment

A total of 36 rats were randomly divided into three groups: paeonol group, vehicle group, and sham group. Rats in the paeonol group were pre-administered paeonol one hour before introduction of tMCAo. A dose of 20 mg/kg (i.p.) was chosen according to our previous study, which revealed that 20 mg/kg of paeonol exhibits the best neuroprotective effect [14]. After 10 min of ischemia, rats were subjected to reperfusion. Rats in the vehicle group were subjected to the same procedure as rats in the paeonol group, but PBS was administered instead of paeonol. Rats in the sham group underwent the same procedure as rats in the vehicle group, but the origin of the MCA was not occluded.

One day before tMCAo, rats underwent habituation and training for the passive avoidance trial. One hour before tMCAo, rats underwent the retention trial. Twenty-four hours after tMCAo, the neurological status were measured. The second retention trial and evaluation of neurological status were performed on the seventh day after tMCAo. Rats were then sacrificed for Western blotting and terminal deoxynucleotidyl transferase-mediated dUTP-biotin nick end labeling (TUNEL) and immunohistochemical (IHC) staining.

### 2.4. Measurement of Neurological Status

The neurological status of each rat was measured using Modified Neurological Severity Score, 24 hours after reperfusion, by an investigator blind to the treatment group. Motor, sensory, balance, and reflex functions were assessed based on a neurological deficit score (18-point scale) described by Chen et al. [17]. Briefly, motor tests included placing the rat on the floor (inability to walk straight was scored as 1, circling toward paretic side was scored as 2, and falling down to the paretic side was scored as 3) and raising each rat by its tail (flexion of forelimb was scored as 1, flexion of hindlimb was scored as 1, and head moving >10° was scored as 1). Sensory tests included tactile (deficiency, 1) and pushing paw against table edge (deficiency, 1) subtests. Ability to balance on the beam was scored as follows: rats grasped onto side of a beam, 1; hugged the beam and one limb slipped off the beam, 2; hugged the beam and two limbs slipped off the beam, 3; attempted to balance but fell off (>40 s), 4; attempted to balance but fell off (<20 s), 5; fell off the beam without any attempt to balance, 6. Reflex tests included pinna reflex (deficiency, 1), corneal reflex (deficiency, 1), and startle reflex subtests (deficiency, 1). Abnormal movement was scored (seizure, 1).

### 2.5. Passive Avoidance Test

The passive avoidance apparatus consisted of two chambers of the same size (25 × 20 × 17 cm high) connected via a guillotine door (9 × 7 cm). The floor of each chamber was made of 14 stainless steel rods (6 mm in diameter), spaced 1.8 cm center to center, and wired to a shock scrambler (Gemini Avoidance System, San Diego Instruments, San Diego, CA, USA). For habituation the rats were placed in the right chamber of the apparatus and, 5 sec later, the house light was turned on and the guillotine door was raised. Upon entering the dark chamber, the guillotine door was closed and 30 sec later the rats were taken out from the dark chamber and put into their home cages. Entrance latency into the dark compartment was recorded when the animal had placed all four paws into the dark chamber. If the animal waited for more than 100 sec to enter the dark chamber, it was eliminated from the experiment. The habituation was repeated 30 min later and followed after the same interval by a training session. During the training session the guillotine door was closed and an intermittent electric shock (50 Hz, 3 s, 0.5 mA) was delivered to the floor of the dark chamber immediately after the animals had enter the dark chamber. After 30 sec, the rat was taken from the dark chamber and placed into its home cage. Then after 2 min, the training session was repeated. The rat received a footshock each time it reentered the dark. Training was terminated when the rat remained in the light compartment for 120 sec. The numbers of trials (entering the dark chamber) were recorded. The retention trial was performed 24 hours and seven days after tMCAo. After the house light was on and the guillotine door was open, the step-through latency (STL) into the dark chamber was recorded for up to 300 sec. If the rat did not enter the dark chamber after 300 sec, the retention trial was terminated and a ceiling score of 300 sec was assigned.

### 2.6. IHC Assay

For ICH and TUNEL staining, rats were transcardially perfused with 200 mL of 0.9% saline and 200 mL of 4% paraformaldehyde (PFA, pH 7.4). Rat brains were removed quickly and postfixed in 4% PFA followed by 30% sucrose  (wt/vol) for three days and then cut into 15 *μ*m sections using a cryostat. Brain sections were rinsed with Dulbecco's phosphate-buffered saline (DPBS, Sigma, USA) containing 0.01% Tween-20 and immersed in 3% H_2_O_2_/methanol for 15 min to inhibit endogenous peroxidase activity. Thereafter, sections were incubated with 10% normal animal serum (Zymed, CA, USA) for 20 min at room temperature. The sections were incubated in moist chambers with primary anti-APP (1 : 100 dilution, mouse monoclonal, 22C11, Chemicon, Billerica, MA, USA) and anti-BACE (1 : 100 dilution, Chemicon) for one hour at 37°C. Following incubation with secondary antibody and avidine-biotin peroxidase complex (ABC kit, Zymed, CA, USA) sections were colored using a 3,3′-diaminobenzidine (DAB) kit (Scytek Laboratories, Logan, U.T, USA), and then counterstained with hematoxylin. The stained sections were mounted in mounting media (Assistant-Histokitt, Germany) and immunoreactive cells were counted under the microscope (Axioskop 40, Zeiss). Immunoreactive cells were counted for nine consecutive high power fields (HPFs) along the CA1 region and nine HPF within a square in the MCA territory of the cortex (Figure 3(a)). Negative control stains were performed on adjacent sections in the control group and subjected to the same IHC assay procedures, but without primary antibodies.

### 2.7. TUNEL Assay

TUNEL staining was performed according to the manufacturer's instructions of a commercial kit (Merck KGaA, Darmstadt, Germany) to identify cells with nuclear DNA fragmentation. Briefly, the brain sections, which were chosen adjacent to those used for IHC were incubated with proteinase K (20 *μ*g/mL) for 20 min and then incubated with 1X TdT equilibration buffer for 30 min at room temperature, followed by incubation with TdT labeling reaction mixture for 1.5 h at 37°C. After addition of stop solution and blocking buffer, sections were incubated with conjugate solution for 30 min at room temperature and TUNEL positive cells were visualized using DAB kit. Finally, sections were counterstained with methyl green. 

### 2.8. Western Blotting Analysis

For Western blotting, rats were anesthetized using choral hydrate and perfused transcardially with 400 mL of 0.9% saline. Brains were removed, coronally sectioned from −4.3 to +1.7 mm bregma, and separated into the right cortex, right striatum, left cortex, and left striatum. The right cortex was weighed and the cytosolic and mitochondrial proteins were separated using a commercial kit (BioVision, Mountain View, CA, USA). Protein concentrations of the cytosolic and mitochondrial fractions were determined using Bio-Rad assay. The protein extracts (10 *μ*g) were then separated using 10% sodium dodecyl sulfate-polyacrylamide gel electrophoresis, transferred to nitrocellulose membranes, probed with antibodies, and detected using peroxidase-conjugated anti-rabbit antibody followed by chemiluminescence as described previously [18]. The antibody against actin (dilution 1 : 5000) was purchased from Chemicon. Antibodies against cytochrome c (1 : 1000 dilution), Bax (1 : 1000 dilution), B-cell leukemia/lymphoma-2 (Bcl-2) (1 : 1000 dilution), and tumor necrosis factor receptor type 1-associated death domain (TRADD) (1 : 1000 dilution) were purchased from Cell Signaling (Beverly, MA, USA). Antibodies against apoptosis-inducing factor (AIF) (1 : 100 dilution) and Fas-associated death domain (FADD) (1 : 1000 dilution) were purchased from Calbiochem (San Diego, CA, USA), antibody against cleaved caspase-8 (1 : 1000 dilution) was purchased from BioVision (Mountain View, CA, USA), and that against Cox4 (1 : 5000) was purchased from Abcam (Cambridge, MA, USA). The intensities of bands on the gel were calculated using Gel-Pro Analyzer (Media Cybernetics Inc., Bethesda, MD, USA).

### 2.9. Statistical Analysis

Data are expressed as mean ± SD. Data of sham, vehicle, and paeonol groups were compared using one-way ANOVA followed by posthoc Scheffe's test. A probability value of less than 0.05 was considered statistically significant.

## 3. Results

### 3.1. Effects of Paeonol on Neurological Deficit Induced by tMCAo

**Figure 1:**
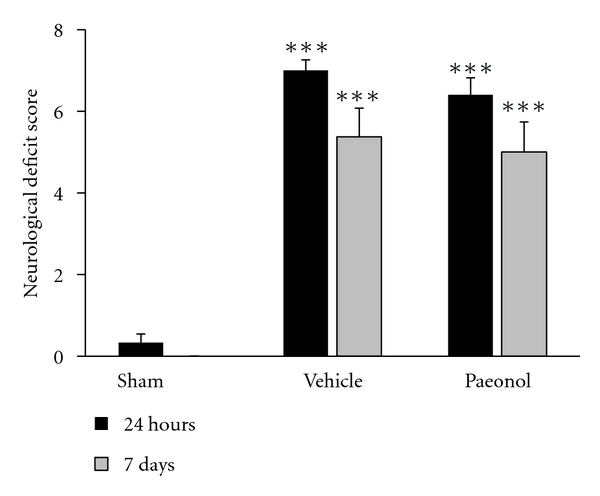
Effects of paeonol on neurological defect 24 hours and seven days after tMCAo. Seven days after tMCAo, rats presented with a total neurological deficit score of 18 points. 13–18 represents severe injury; 7–12 represents moderate injury; 1–6 represents mild injury. ***P < 0.001 compared to sham group (*n* = 12).

There were three groups of rats: sham, vehicle, and paeonol. Rats in the vehicle and paeonol groups received tMCAo via a 10 min occlusion of the MCA, while rats in the sham group received a sham operation. Rats in the paeonol group were pre-administered paeonol one hour before tMCAo. Twenty-four hours after tMCAo, rats in the vehicle and paeonol groups presented neurological deficit scores of 7.0 ± 0.8 (range, 6–8) and 5.5 ± 2.5 (range, 5–8), respectively. Seven days after tMACo the neurological deficit score was 5.4 ± 2.0 in the vehicle group and 4.3 ± 2.7 in the paeonol group. There were no significant differences in neurological deficit score between vehicle and paeonol groups either 24 hours or seven days after tMCAo (Figure 1).

### 3.2. Effects of Paeonol on STL in the Passive Avoidance Test

**Figure 2:**
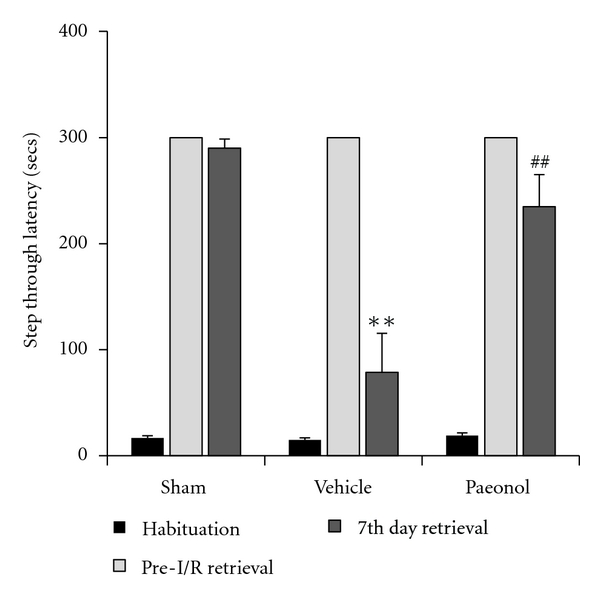
Effects of paeonol on step-through latency (STL) in retention trial seven days after tMCAo. Paeonol (20 mg/kg) was administered 1 h before tMCAo. **P < 0.05 compared to sham group; ^##^ *P* < 0.05 compared to vehicle group (*n* = 12).

In the passive avoidance test, rats were shocked in order to train them to avoid going into the dark chamber 24 hours before tMCAo. Performance of retention trials immediately before tMCAo ensured the rats had remembered the shock from 24 hours previously. Then, repeat of the retention trials seven days after tMCAo tested if the rats had remembered the shock one week previously. Data revealed that the STL into the dark chamber was significantly shorter in the control group than in the sham group seven days after tMCAo. Paeonol pretreatment significantly reversed the decrease in STL induced by tMCAo (*P* = 0.007) (Figure 2).

### 3.3. Paeonol Reduced the Expression of APP and BACE in the Ischemic Brain Seven Days after tMCAo

**Figure 3:**
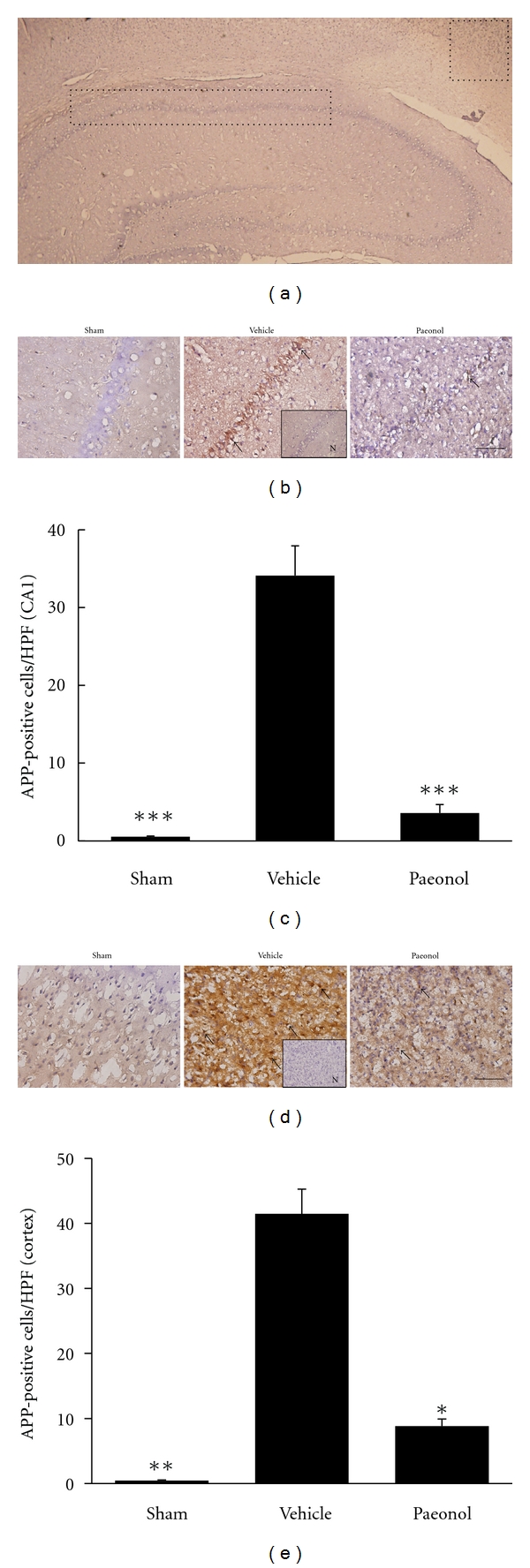
APP immunohistochemistry in the hippocampus and ischemic cortex seven days after tMCAo. (a) The region within the rectangular dotted line indicates the CA1 sector, and the region within the square dotted line indicates the MCA territory of cortex, for calculation of APP-immunopositive cells. (b) Representative photographs showing APP immunoreactivity in the hippocampal CA1 area. (c) APP-positive cells per 400X microscopic field in the CA1 area. (d) Representative photographs showing APP immunoreactivity in the cortex. (e) APP-positive cells per 400x microscopic field in the cortex. In (b,d), scale bar represents 100 *μ*m. Arrows indicate APP-immunoreactive cells. In (c,e), *P < 0.05, **P < 0.01, and ***P < 0.001, refer to the differences from the vehicle group. Error bars denote S.E.M (*n* = 6).

**Figure 4:**
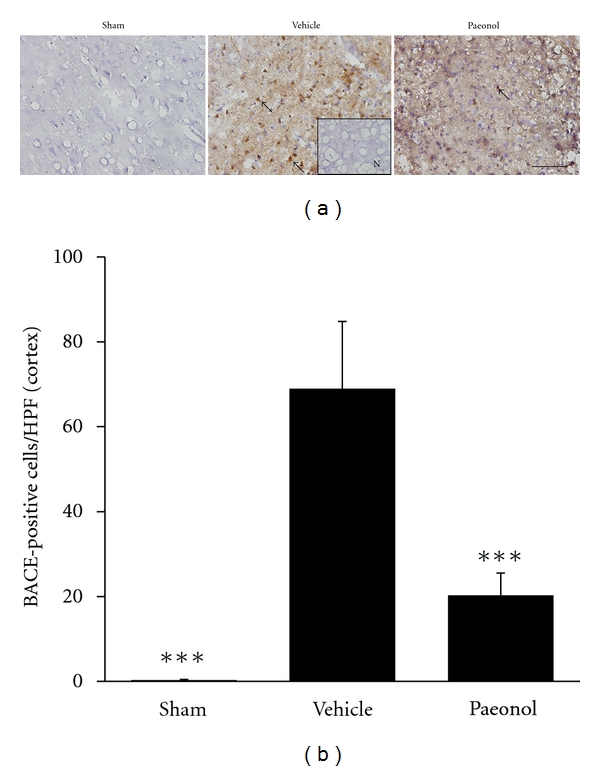
BACE immunohistochemistry in the ischemic cortex seven days after tMCAo. (a) Representative photographs showing BACE immunoreactivity in the cortex. Scale bar represents 100 *μ*m. Arrows indicate BACE-immunoreactive cells. (b) BACE-positive cells per 400x microscopic field in the cortex. Error bars denote SEM. In (b), ***P < 0.001, refers to the differences from the vehicle group (*n* = 6).

IHC staining evaluated APP- and BACE-expressing cells within the dotted line areas of the brain coronal section (Figure 3(a)). On day seven after tMCAo, the sham group presented minimal APP and BACE immunoreactivity. The CA1 area of hippocampus and MCA territory of the cortex in the vehicle group demonstrated increased APP immunoreactivity. Rats in the paeonol group demonstrated smaller increases in APP immunoreactivity in the CA1 and cortical areas than those in the vehicle group (Figures 3(b), 3(c), 3(d), and 3(e)). tMCAo induced increases in the numbers of BACE-immunoreactive cells in the MCA territory of the cortical region. However, the paeonol group demonstrated reduced BACE immunoreactivity (Figures 4(a) and 4(b)). The CA1 area contained no BACE immunoreactive cells in the sham, vehicle, and paeonol groups. These data suggest that paeonol suppressed tMCAo-induced APP in the CA1 and cortex and also suppressed BACE expression in the cortex. 

### 3.4. Effects of Paeonol on Apoptosis Seven Days after tMCAo

**Figure 5:**
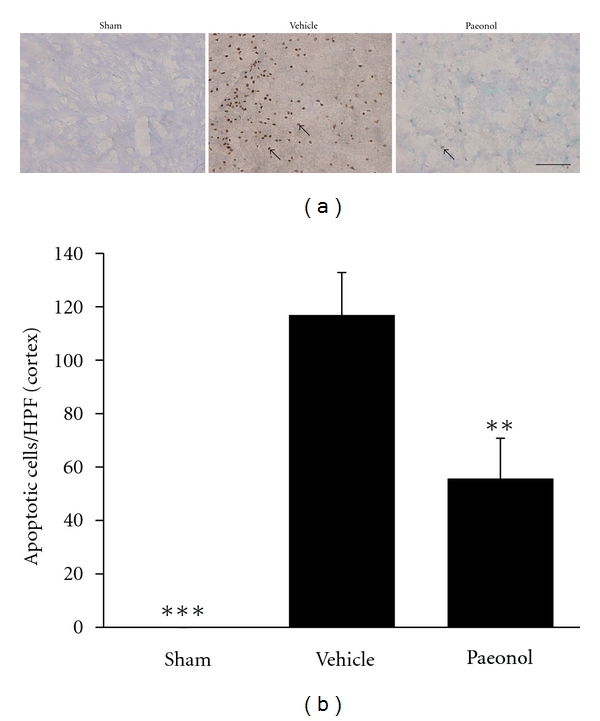
Effects of paeonol on apoptosis in the ischemic cortex seven days after tMCAo. (a) Representative photographs showing TUNEL staining in the ischemic cortex. Scale bar represents 100 *μ*m. Arrows indicate TUNEL-positive cells. (b) TUNEL-positive cells per 400x microscopic field in the cortex. Error bars denote S.E.M. **P < 0.01 and ***P < 0.001, refer to the differences from the vehicle group (*n* = 6).

TUNEL staining detected apoptotic cells seven days after tMCAo. There were no TUNEL-positive cells in the cortical area in the sham group (Figures 5(a) and 5(b)). The number of TUNEL-positive cells in the MCA territory of the cortical region in the vehicle group substantially increased seven days after tMCAo. In contrast, the paeonol group demonstrated marked reductions in TUNEL-positive cells compared to the vehicle group. Cell counts showed that paeonol reduced the number of TUNEL-positive cells to 52.3% (Figure 5(b)). There were no TUNEL-positive cells in the CA1 region in sham, vehicle, or paeonol groups.

**Figure 6:**
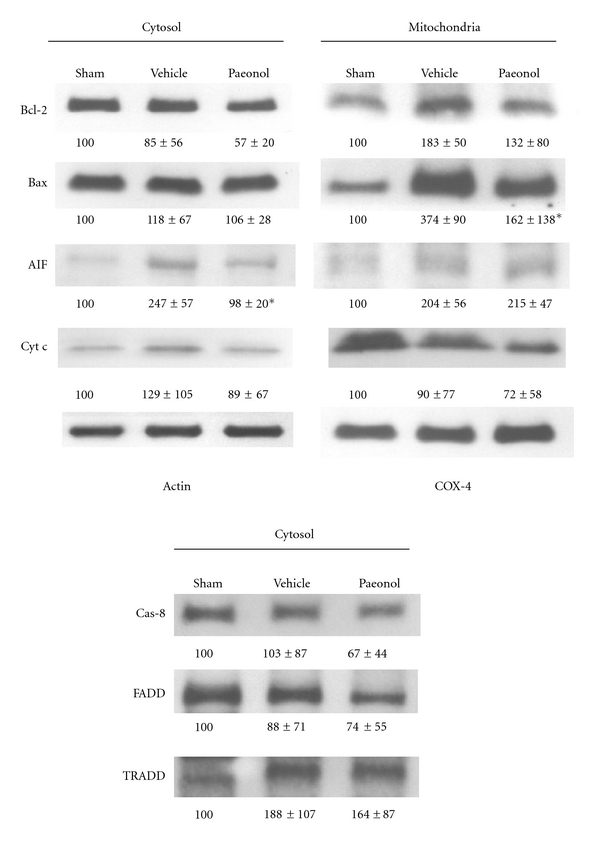
Western blot analysis of apoptosis-related proteins from cytosolic and mitochondrial fractions. Data are shown as mean ± SEM of five independent assays. The percentage below each lane represents the amount of protein relative to the sham group. *P < 0.05 compared with the vehicle group.

 To determine the possible pathways in which paeonol might participate to suppress apoptosis, the present study extracted cytosolic and mitochondrial proteins from the ischemic cortex for Western blot analysis. Of the apoptosis-related proteins examined, tMCAo increased AIF by more than twofold in both cytosolic and mitochondrial fractions. Paeonol suppressed this increase in AIF in the cytosol. In mitochondria, levels of AIF protein in the paeonol group did not differ from those in the vehicle group. tMCAo induced 3.74-fold increases in mitochondrial Bax protein while paeonol treatment reduced the induced Bax to 1.62-fold of its baseline value. Administration of paeonol did not alter cytosolic Bcl-2, Bax, cytochrome c, caspase-8, FADD, and TRADD protein levels and also did not modify mitochondrial Bcl-2 and cytochrome c (Figure 6).

## 4. Discussion

Based on previous studies which identified that paeonol improves cognitive functions and inhibits APP expression [13, 15], the present study examined paeonol's potential memory protective effects in a cerebral ischemic rat model. Data demonstrated that paeonol protected memory in a subacute stage seven days after tMCAo. The possible molecular mechanisms included the reduction of *β*-amyloid levels and the suppression of apoptosis. Procedures also identified Bax and AIF as the key molecules suppressed by paeonol during the apoptotic process.

Prior research has identified that paeonol exerts neuro-protective effects and reduces the infarct area in cerebral artery occlusion and cerebral ischemia-reperfusion models [17, 19, 20]. The protection of paeonol against neuronal damage is previously ascribed to its antioxidative activities. In one study, paeonol increased superoxide dismutase and glutathione activities [13]. In another study, paeonol attenuated hydrogen peroxide-induced transcription factor NF-*κ*B [15], which is highly relevant in the inflammatory process following ischemic stroke [21]. Zhong et al. also attributed the improvements to cognitive impairment induced by d-galactose, which generates superoxide anion and oxygen-derived free radicals, to the antioxidative ability provided by paeonol [13].

In this study, paeonol had no significant effects on neurological deficit on the first or seventh day after reperfusion, conflicting with our previous study that showed paeonol reduced the neurological deficit scores. The inconsistency might come from the different neurological deficits generated by different ischemic time periods in the two studies. In that previous study, the rats underwent tMCAo that blocked MCA for 90 minutes before reperfusion, generating a severe injury with the neurological deficit score more than 13 [14, 20]; however, rats hardly survived more than 48 hours. In this study, in order to examine memory in the subacute stage of ischemic stroke, we abandoned the severe deficit model and adopted moderate deficit model that blocked the blood flow for 10 minutes, generating the neurological deficit score of 7.0 ± 0.8 (range, 6–8). We proposed that it was because that the neurological deficit scores were smaller in this study than that in our previous one, the data could not show a statistical significance, even the mean neurological deficit score was lower in paeonol group. Although paeonol did not significantly protect neurological functions in this model, paeonol reversed the tMCAo-induced decreases in STL in a passive avoidance retrieval test, indicating that paeonol protected memory after cerebral ischemia.

In previous studies, paeonol improved learning ability and enhanced memory in a D-gal-injured brain, tested using the Morris water maze and passive avoidance tests [13]. Paeonol also altered behaviors of learning in rats following *β*-amyloid injection [22]. Amyloidopathy is one of the main mechanisms of pathogenesis in poststroke dementia. A number of prior investigations observed upregulation of APP and *β*-amyloid following cerebral ischemia, mainly in astrocytes of the hippocampal CA1 area [23–25], and also in the cortex and corpus callosum one to four weeks after ischemia-reperfusion, leading to dense plaque-like formation after a nine month followup [4]. The present study observed increased APP in hippocampal and cortical areas seven days after tMCAo, and attenuation of APP increases in both regions following paeonol treatment. However, the role of APP in the central nervous system remains controversial. One of the APP products is the deleterious *β*-amyloid, while another APP product, sAPP*α*, may participate in neuroprotection, synaptic plasticity, neurite outgrowth, and synaptogenesis [26]. Increased APP expression might also participate in the protection against endoplasmic reticulum stress [27]. Base on our data, we speculated that APP was not the key factor for neuronal damage seven days after tMCAo, because there was no apoptosis and no infarction observed in hippocampus (data not shown), even APP level was up-regulated in the same area. On the other hand, the coinduction and colocalization of BACE with apoptosis after tMCAo in cortex suggested that BACE played a more deleterious role than APP.

The three types of proteases which cleave APP to generate *β*-amyloid are *α*-, *β*-, and *γ*-secretase [28]. Among the three secretases, BACE coexpresses most highly with *β*-amyloid [29, 30], and controls the rate-limiting step in the production of *β*-amyloid [31]. The high correlation between BACE and *β*-amyloid renders BACE being considered one of the effective therapeutic targets to treat *β*-amyloid-related diseases [32, 33]. In previous studies, focal ischemia increased the activity of BACE, especially BACE1, in the ischemic cortex [7] and thalamus [34]. Consistent with a previous study by Wen et al. [7], the present study observed BACE induction and apoptosis occurred in the same location, that is, ischemic cortex, suggesting the high relevance of BACE and apoptosis in ischemic stroke. Paeonol treatment limited the induction of BACE and suppressed apoptosis, indicating that suppression of *β*-amyloid production by paeonol might closely link to the suppression of apoptosis, and might participate in the memory protection after cerebral ischemia.

After cerebral ischemia, apoptosis occurs in CA1 within three days of cerebral ischemia, disappears after seven days, and do not reoccur after a further seven days [35, 36]. In the present study, apoptosis was observed in the ischemic cortex, but not in the CA1 region, seven days after tMCAo. The suppression of apoptosis in the ischemic cortex indicated that paeonol protected neuronal cells from tMCAo injury. In recent research, paeonol also has been shown to inhibit glutamate-induced apoptosis in PC12 cells [37] and in rat brains receiving *β*-amyloid injection [22], while the detailed mechanisms relating to the suppression of apoptosis in neuronal cells have yet to be identified. There are two general pathways of apoptosis following cerebral ischemia: the intrinsic and extrinsic pathways. The intrinsic pathway is initiated by the accumulation of intracellular Ca^2+^ following the depletion of energy after ischemia [10]. Activation of calpains by Ca^2+^ results in cleaving of the Bcl-2 interacting domain (BID) to its truncated form (tBID), which targets the outer mitochondrial membrane and induces conformational changes in proapoptotic proteins, such as Bak, Bax, Bad, and Bcl-XS. These pro-apoptotic proteins can also heterdimerise with antiapoptotic proteins, including Bcl-2 or Bcl-XL [38]. The binding of tBID to pro-apoptotic proteins opens the mitochondrial transition pore, leading to the release of cytochrome c and AIF into the cytosol [39]. Once released from mitochondria, cytochrome c binds to and activates caspase-9, and then caspase-3, which cleaves poly (ADP-ribose) polymerase (PARP), leading to DNA injury and caspase-dependent apoptotic cell death [40]. Cytosolic AIF further translocates into the nucleus and stimulates caspase-independent DNA fragmentation [41]. The extrinsic pathway starts from the activation of the cell surface Fas and tumor necrosis receptors. The Fas receptor triggers FADD directly and tumor necrosis receptor triggers FADD via TRADD, activating caspase-8, and then caspase-3, resulting in PARP cleavage and DNA damage [42]. The present study is the first to identify Bax and AIF as the two main molecules modified by paeonol which paeonol can adjust during the suppression of apoptosis following cerebral ischemia.

The reduction of Bax by paeonol suggests that paeonol might suppress the apoptosis by inhibiting the release of mitochondrial factor into cytosol. The simultaneous suppression of cytosolic AIF supported this assumption. However, level of cytochrome c was not significantly changed by paeonol, although the mean value of cytochrome c was lower in the paeonol group (0.89-fold sham) than that in the vehicle group (1.29-fold sham). We proposed that the small enhancement of cytochrome c decreased the statistical significance. A report by Zhu et al. described that after cerebral ischemia, AIF expression was more pronounced in neurons of the male brain, while female brain neurons showed marked increases in expression of caspase-3 [43]. The present study with male rats induced a 2.47-fold of elevated AIF in cytosol, which was reversed to almost baseline levels. There are only a few herbs which are known to regulate the expression of AIF. *Isatis indigotica* [44] and polyphyllin D [45] increase the release of AIF from mitochondria, causing apoptosis, while berberine inhibits AIF and therefore suppresses apoptosis [46].

Medicinal plant extracts and natural antioxidants might have potential use in the prevention and treatment of dementia [2, 47]. Results from the present study indicate that paeonol, which derives from a widely administered medicinal herb, protects the memory following cerebral ischemia. Paeonol treatment reduced APP and BACE expression, and also the numbers of apoptotic cells. Suppression of the level of Bax protein and blocking AIF from releasing to the cytosol might also be the mechanisms in which paeonol exerted its effect on anti-apoptosis and memory protection.

## Abbreviations

AIF: Apoptosis-inducing factor
APP: Amyloid precursor protein
BACE: Beta-site APP cleaving enzyme
Bcl-2: B-cell leukemia/lymphoma-2
CCA: Common carotid artery
DAB: 3,3′-Diaminobenzidine
HPF: High power field
ICA: Internal carotid artery
IHC: Immunohistochemistry
MCA: Middle cerebral artery
tMCAo: Transient middle cerebral artery occlusion
PBS: Phosphate-buffered saline
STL: Step-through latency
TUNEL: Terminal deoxynucleotidyl transferase-mediated dUTP-biotin nick end labelling.
